# Metaphorical perceptions of secondary school students regarding the concept of generative artificial intelligence

**DOI:** 10.1007/s00431-026-07007-0

**Published:** 2026-05-02

**Authors:** Özkan Özbay

**Affiliations:** Distance Education Application and Research Center, Artvin Çoruh University, Artvin, Türkiye

**Keywords:** Generative artificial intelligence, Metaphor analysis, Secondary school students, AI literacy, Educational technology

## Abstract

The rapid integration of generative artificial intelligence (GenAI) tools, such as ChatGPT, into educational contexts has raised important questions regarding how adolescents conceptualize and make sense of these technologies. Understanding students’ perceptions is essential for developing age-appropriate, ethical, and pedagogically sound approaches to AI use in secondary education. This descriptive qualitative study employed a phenomenological approach and metaphor analysis to explore secondary school students’ perceptions of generative artificial intelligence. The study sample consisted of 332 students aged 14–18 years from four secondary schools in Türkiye. Data were collected using an open-ended prompt (“Generative artificial intelligence is like … because …”) and analyzed through content analysis. Metaphors were categorized based on shared semantic and conceptual features, and inter-rater reliability was established using Cohen’s kappa (*κ* = 0.92). Analysis revealed ten metaphor categories clustered under five overarching themes: generative artificial intelligence as (1) a source of knowledge, (2) a teaching and guiding entity, (3) a supportive and assisting tool, (4) a reflection of human intelligence, and (5) a dual-purpose (beneficial–risky) technology. Students most frequently conceptualized GenAI as a comprehensive knowledge source (e.g., book, encyclopedia) and as a human-like cognitive entity (e.g., brain, wise person). At the same time, metaphors reflecting ethical awareness and potential risks, such as misuse and overreliance, were also identified. The findings indicate that secondary school students hold multifaceted and nuanced perceptions of generative artificial intelligence, encompassing both educational opportunities and ethical concerns. These results highlight the importance of integrating AI literacy into secondary education in ways that promote critical thinking, responsible use, and awareness of GenAI’s limitations alongside its potential benefits.

*Conclusion*: It was determined that secondary school students perceive generative artificial intelligence ambivalently as both a useful tool and a source of ethical and emotional concern, highlighting the need for developmentally appropriate artificial intelligence literacy approaches.

**Table:** 

**What is Known:** *• GenAI tools such as ChatGPT are increasingly integrated into educational contexts and have the potential to support personalized learning, information access, and student engagement.* *• Existing research has primarily focused on educators’ perspectives or higher education settings, while studies examining adolescents’ perceptions of GenAI remain limited.*	
**What is New:** *• This study provides empirical evidence on secondary school students’ metaphorical perceptions of generative artificial intelligence within a K–12 context.* *• Findings reveal that adolescents conceptualize GenAI in multifaceted ways, including as a knowledge source, teaching and guiding entity, supportive tool, reflection of human intelligence, and a dual-purpose (beneficial–risky) technology.*

**What is Known:**

*• GenAI tools such as ChatGPT are increasingly integrated into educational contexts and have the potential to support personalized learning, information access, and student engagement.*

*• Existing research has primarily focused on educators’ perspectives or higher education settings, while studies examining adolescents’ perceptions of GenAI remain limited.*

**What is New:**

*• This study provides empirical evidence on secondary school students’ metaphorical perceptions of generative artificial intelligence within a K–12 context.*

*• Findings reveal that adolescents conceptualize GenAI in multifaceted ways, including as a knowledge source, teaching and guiding entity, supportive tool, reflection of human intelligence, and a dual-purpose (beneficial–risky) technology.*

## Introduction

The release of ChatGPT in late 2022 led to a marked global increase in interest in generative artificial intelligence (GenAI) [1]. Subsequently, a wide range of GenAI tools, including text-based chatbots and image-generation systems, have rapidly become embedded in educational contexts [2–4]. Existing research indicates that GenAI has the potential to support personalized learning and enhance student engagement, positioning education as a key domain for AI-driven transformation [5–7]. However, the literature also highlights significant concerns, including overreliance on AI, the dissemination of inaccurate information, privacy and copyright issues, and inequalities in access [1, 8]. In this context, understanding how students conceptualize generative artificial intelligence within their learning and everyday lives is critical for developing safe, ethical, and pedagogically responsible approaches to its use.

One of the most effective approaches to examining students’ conceptualizations of complex and emerging technologies is metaphor analysis. Grounded in Conceptual Metaphor Theory, metaphors function as fundamental cognitive tools that enable individuals to interpret abstract phenomena through familiar experiences [9]. Accordingly, metaphors extend beyond linguistic expressions and serve as indicators of individuals’ mental representations, beliefs, and value orientations [10]. In educational research, metaphor analysis has been widely used to explore how learners and educators perceive emerging technologies, often framing them as sources of knowledge, guiding agents, supportive tools, or potential threats [11, 12]. Within the context of artificial intelligence, this approach provides a nuanced and human-centered perspective for understanding both the perceived benefits and ethical concerns associated with GenAI [13, 14].

Recent studies have demonstrated that students conceptualize artificial intelligence through diverse and multidimensional metaphorical frameworks, including representations as knowledge sources, human-like entities, and dual-purpose technologies encompassing both opportunities and risks [15, 16]. These findings suggest that students’ perceptions of AI are shaped not only by cognitive interpretations but also by emotional and ethical considerations. However, despite the increasing number of such studies, the literature remains limited in analytical depth and lacks a comprehensive synthesis of findings across different educational levels. Existing studies are often examined in isolation, which restricts the development of a coherent and integrative understanding of students’ perceptions of artificial intelligence.

Furthermore, the theoretical positioning of prior research has frequently remained implicit, with limited engagement with broader conceptual frameworks that explain how these perceptions are constructed and interpreted [9, 17]. This limitation highlights the need for studies that explicitly integrate metaphor-based approaches within established theoretical perspectives. In this regard, Conceptual Metaphor Theory provides a robust framework for interpreting students’ cognitive and affective meaning-making processes, while its integration with educational technology perspectives enables a more comprehensive understanding of how emerging technologies are experienced in learning environments [17–19].

Despite the growing body of research on artificial intelligence in education, an important gap remains regarding secondary school students. Most existing studies have focused on either primary school contexts or higher education populations, with relatively limited attention given to adolescents [8, 20, 21]. This gap is particularly critical, as adolescence represents a developmental stage characterized by the advancement of abstract thinking, the emergence of ethical awareness, and increased engagement with digital technologies. Therefore, examining how secondary school students conceptualize generative artificial intelligence is essential for developing age-appropriate and pedagogically effective AI literacy initiatives.

Addressing this gap, the present study aims to explore secondary school students’ metaphorical perceptions of generative artificial intelligence within the context of Türkiye. By systematically analyzing the metaphors produced by students, the study seeks to provide a multidimensional understanding of how GenAI is positioned as a cognitive tool, a human-like entity, and a technology associated with both opportunities and risks. In doing so, the study contributes to the literature by offering a more theoretically grounded and analytically integrated perspective on students’ perceptions of artificial intelligence, while also providing practical implications for the development of AI literacy and educational integration strategies. 

**Research questions**

1. Which metaphors do secondary school students use to describe the concept of “generative artificial intelligence”?
2. What underlying perceptions and meaning-making patterns regarding generative artificial intelligence are reflected in the metaphors used by students?
3. How do these metaphors reveal the ways in which students position generative artificial intelligence in relation to the learning process?

## Methods

### Study design

This study is a descriptive qualitative inquiry grounded in a phenomenological approach and employs metaphor analysis as the primary analytical technique. Metaphor analysis is situated within the phenomenological research tradition and enables the exploration of individuals’ perceptions of abstract and complex concepts through their associations with familiar experiences [22].

Metaphors are widely recognized in qualitative research as a powerful means of revealing individuals’ mental representations and meaning-making processes [23]. In this study, secondary school students’ perceptions of generative artificial intelligence were examined through the metaphors they produced, and the meanings they attributed to this concept were descriptively analyzed based on participants’ own expressions.

### Study sample

The study population consisted of students enrolled in four different secondary schools located in the city center of Artvin. The study sample was determined based on voluntary participation and comprised 332 secondary school students aged between 14 and 18 years, representing different grade levels. Inclusion criteria for participation were being enrolled in one of the selected secondary schools, being within the specified age range, having the cognitive ability to comprehend and respond to the data collection form, and providing voluntary consent to participate. Data were collected by the researchers through face-to-face interviews conducted in classroom settings within the school environment.

A convenience sampling strategy was employed, and a total of 346 students initially participated in the study. During the data-cleaning process, responses that did not contain a clearly identifiable metaphor, lacked a meaningful relationship between the metaphor and its justification, or included incomplete or insufficient “because” statements were excluded from the analysis. A total of 14 responses were excluded based on these criteria. Consequently, 332 valid metaphors produced by 332 participants were included in the final analysis.

### Data collection procedure

The study data were collected through metaphors, a qualitative data collection technique widely used to explore and interpret individuals’ experiences and perceptions. The use of metaphors enables an in-depth understanding of how individuals perceive abstract concepts by relating them to familiar experiences and articulating the meanings they attribute to these associations [23].

Data were collected between November and December 2025 using a data collection form developed by the researchers. The instrument consisted of two sections. The first section included questions aimed at identifying students’ demographic characteristics, such as age, gender, and grade level. The second section asked students to generate a metaphor related to the concept of generative artificial intelligence. For this purpose, students were provided with the following open-ended prompt:

“Generative artificial intelligence is like … because …”

Students were asked to compare generative artificial intelligence to an object, entity, or concept and briefly explain the reason for their comparison. The handwritten responses produced by the students constituted the primary data source of the study.

### Ethical considerations

Ethical approval for the study was obtained from the Scientific Research and Publication Ethics Committee of Artvin Çoruh University (Approval No.: E-18457941–050.99–193,606), and official permission to conduct the research in schools was granted by the Provincial Directorate of National Education (Approval No.: MEB.TT.2025.029901.03). All procedures were conducted in accordance with the principles of the Declaration of Helsinki. Written informed consent was obtained from both students and their parents prior to participation. Participation was voluntary, and students were informed of their right to withdraw from the study at any time without any consequences. Data were collected anonymously, no personally identifiable information was recorded, and all data were securely stored on password-protected computers accessible only to the research team.

### Data analysis

Qualitative data were analyzed using content analysis, a method aimed at systematically organizing participants’ metaphors into meaningful conceptual patterns and interpreting similarities and differences within the data [23, 24]. Content analysis enables the transformation of qualitative expressions into structured categories while preserving the underlying meanings conveyed by participants.

The analysis was conducted in five sequential stages, consistent with procedures outlined in the literature [25]. In the naming stage, all metaphors generated by students were listed, and responses that did not include a clear metaphor or justification were excluded from further analysis. During the classification stage, metaphors produced in response to the prompt “Generative artificial intelligence is like … because …” were carefully reviewed and grouped based on shared characteristics.

In the categorization stage, metaphors were organized into conceptual categories based on their common semantic and analogical characteristics. Particular emphasis was placed on students’ explanations following the word “because” to ensure that both the metaphor and its intended meaning were adequately represented. At this stage, the data were first subjected to an open coding process, in which each metaphor and its accompanying justification were treated as distinct meaning units. Subsequently, a constant comparative method was employed to identify recurring patterns and relationships across the data. Metaphors were grouped under the same category when they reflected a similar underlying perception (e.g., knowledge provision, guidance, assistance, or risk), even if their surface expressions differed. The categorization criteria were guided by (a) the core meaning expressed in the justification, (b) the functional role attributed to generative artificial intelligence (e.g., informational, instructional, or supportive), and (c) the evaluative orientation of the metaphor (e.g., positive, negative, or dual-purpose). Based on these criteria, a coding framework (codebook) was developed to ensure consistency, transparency, and replicability in the analytical process. The categorization procedure was conducted iteratively, with categories continuously refined through comparison with the data until conceptual coherence and internal consistency were achieved.

To ensure analytic rigor and trustworthiness, several strategies were employed. Credibility was enhanced through prolonged engagement with the data and careful consideration of participants’ original expressions. Dependability was supported by maintaining a transparent and systematic coding procedure, including the development and refinement of a codebook. Confirmability was strengthened by grounding all interpretations in participants’ statements and minimizing researcher bias through iterative comparison. Additionally, the involvement of independent field experts contributed to the validation of the analytical structure.

To enhance the reliability of the analysis, the finalized metaphor list and category structure were independently reviewed by two field experts. Inter-rater agreement was assessed using Cohen’s kappa, yielding a coefficient of 0.92, which indicates a high level of agreement. Expert feedback was incorporated, and the coding framework and category structure were refined accordingly. In the final stage, the data were quantified by calculating the frequency and percentage distribution of metaphors within each category, and the results were presented in tabular form. Descriptive statistics related to participants’ demographic characteristics were analyzed using IBM SPSS Statistics 24.0, including frequencies, percentages, means, and standard deviations.

## Results

### Participant characteristics

A total of 332 secondary school students participated in the study. The mean age of the participants was 15.62 years (SD = 1.06). Of these, 170 (51.2%) were female and 162 (48.8%) were male. In terms of grade-level distribution, 22.4% of students were in the 9th grade, 28.2% in the 10th grade, 24.6% in the 11th grade, and 24.8% in the 12th grade.

### Metaphorical findings

**Fig. 1:**
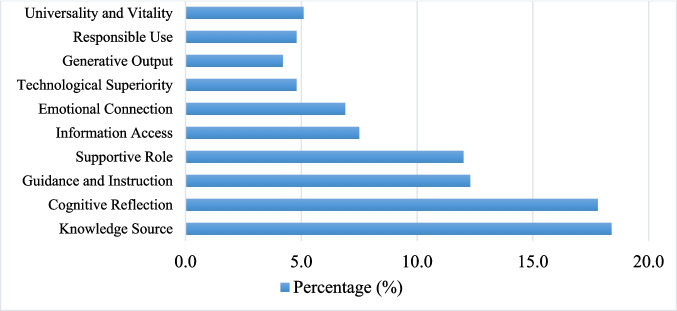
Percentage distribution of metaphor categories

The analysis of students’ metaphorical expressions resulted in the identification of ten conceptual metaphor categories. Metaphors with similar meanings were grouped under broader semantic categories to enhance conceptual clarity and consistency. The frequency and percentage of each category are presented in Table 1 and Fig. 1.

**Table 1:** Frequency and percentage distribution of metaphor categories related to generative artificial intelligence (*N* = 332)

Metaphor category	Example sub-concepts	Frequency (*n*)	Percentage (%)
Book/encyclopedia/library/dictionary (knowledge source)	Book, encyclopedia, library, dictionary, knowledge box, source of information	61	18.4
Human/brain/wise person (cognitive reflection)	Human, brain, human brain, wise person, intelligent mind	59	17.8
Teacher/educator (guidance and instruction)	Teacher, professor, advisor, guide, philosopher, virtual teacher	41	12.3
Assistant/helper/secretary (supportive role)	Assistant, personal helper, secretary, coach, homework partner, supportive robot	40	12.0
Technology/tool/search engine (information access)	Google, computer, tool, answer machine, algorithmic system	25	7.5
Friend/social relationship (emotional connection)	Friend, companion, mentor, parent, psychologist	23	6.9
Power/speed/quality (technological superiority)	BMW, Mercedes, cheetah, light, jewel, generator	16	4.8
Creativity/production/enjoyment (generative output)	Artist, painter, pen, dough, imagination, fun use	14	4.2
Dual risk/ethical awareness (responsible use)	Medicine, weapon, trash bin, cheat sheet, servant, limit	16	4.8
World/scale/life necessity (universality and vitality)	World, pocket world, ocean, water, oxygen, essential part of life	17	5.1

Based on the metaphor analysis, students’ perceptions of generative artificial intelligence were classified into ten main metaphor categories. The category with the highest frequency conceptualized generative artificial intelligence as a comprehensive source of knowledge through metaphors such as book, encyclopedia, library, and dictionary (18.4%). This was followed by the human/brain/wise person category (17.8%), which reflected metaphors associated with human cognition and intelligence.

The teacher/educator (12.3%) and assistant/helper/secretary (12.0%) categories included metaphors indicating guidance, support, and assistance in academic and daily tasks. Other categories represented generative artificial intelligence as a technology or search tool (7.5%), a social or emotional reference (6.9%), a symbol of power, speed, or quality (4.8%), a tool for creativity and production (4.2%), a universal or essential element of life (5.1%), and a technology associated with ethical awareness and dual risk (4.8%).

Overall, the distribution of metaphor categories indicates that students described generative artificial intelligence using a variety of functional, cognitive, and relational frames, as well as metaphors reflecting awareness of potential risks associated with its use.

As a result of the analysis, five overarching themes were identified: (1) generative artificial intelligence as a source of knowledge, (2) generative artificial intelligence as a teaching and guiding entity, (3) generative artificial intelligence as a supportive and assisting tool, (4) generative artificial intelligence as a reflection of human intelligence, and (5) generative artificial intelligence as a dual-purpose (beneficial–risky) technology.

### Theme 1. Perception of generative artificial intelligence as a source of knowledge

This theme reflects students’ perception of generative artificial intelligence as a comprehensive and easily accessible source of information that provides rapid access to a wide range of knowledge.

#### Subtheme 1.1. Comprehensive and extensive knowledge repository

Students described generative artificial intelligence as a source that contains a wide range of information and allows easy access to knowledge. GenAI may be interpreted as being perceived as an epistemic authority that centralizes knowledge, which could suggest a tendency toward reliance on readily available information rather than critical evaluation.

Generative artificial intelligence is like an encyclopedia because it contains all kinds of information. (P6)

Generative artificial intelligence is like a library because it includes information on every subject, and we can choose what we need. (P16)

Generative artificial intelligence is like a book because we can find everything we are looking for in it. (P92)

#### Subtheme 1.2. Rapid and convenient access to information

In this subtheme, students emphasized the speed and practicality of accessing information. This emphasis on speed may reflect efficiency-oriented learning and could be associated with a preference for quick answers, which might, in some cases, limit deeper cognitive processing.

Generative artificial intelligence is like a dictionary because it knows the meaning of everything. (P123).

Generative artificial intelligence is like a pocket world because it brings the information we want immediately. (P73) 

Generative artificial intelligence is like a knowledge box because it gathers and presents information when we need it. (P51)

### Theme 2. Perception of generative artificial intelligence as a teaching and guiding entity

This theme indicates that students view generative artificial intelligence as an instructional and guiding agent that supports learning by explaining concepts and directing them toward information.

#### Subtheme 2.1. Teacher and educator

Students frequently used metaphors positioning generative artificial intelligence as an explanatory and instructive entity. GenAI appears to be perceived as an alternative instructional agent, which may have implications for traditional pedagogical roles and human-centered interaction.

Generative artificial intelligence is like a teacher because it gives explanatory answers to questions. (P32)

Generative artificial intelligence is like a professor because it teaches in a knowledgeable way. (P77)

Generative artificial intelligence is like a virtual teacher because it quickly explains what we want to learn. (P271)

#### Subtheme 2.2. Guidance and direction

This subtheme reflected metaphors emphasizing direction and guidance toward information. GenAI may be interpreted as a navigational guide, which could potentially be associated with more guided forms of learning and, in some cases, reduced emphasis on independent knowledge construction.

Generative artificial intelligence is like a guide because it shows us the way when we ask questions. (P75)

Generative artificial intelligence is like a philosopher because it produces logical answers to every question. (P273)

Generative artificial intelligence is like a key because it opens the door to information we do not know. (P173)

### Theme 3. Perception of generative artificial intelligence as a supportive and assisting tool

This theme highlights that students perceive generative artificial intelligence as a functional tool that facilitates academic tasks and provides assistance in both educational and daily-life contexts.

#### Subtheme 3.1. Academic and daily-life assistance

Students described generative artificial intelligence as a tool that facilitates tasks and provides support. GenAI can be interpreted as functioning as a cognitive assistant, which may reflect task delegation and could be associated with changes in independent problem-solving processes.

Generative artificial intelligence is like an assistant because it helps in every area. (P150)

Generative artificial intelligence is like a homework partner because it helps with assignments. (P31)

Generative artificial intelligence is like a secretary because it is smart and fast. (P3)

#### Subtheme 3.2. Emotional and social support

Some students used metaphors reflecting social or relational aspects. Anthropomorphic perceptions may suggest emotional attachment, which could enhance engagement while also potentially leading to increased reliance or misinterpretation of AI capabilities.

Generative artificial intelligence is like a friend because it is always there for us. (P96)

Generative artificial intelligence is like a helpful friend because it gives friendly and accurate answers. (P301) 

Generative artificial intelligence is like a psychologist because it analyzes what we tell it. (P76)

### Theme 4. Perception of generative artificial intelligence as a reflection of human intelligence

This theme represents students’ tendency to associate generative artificial intelligence with human-like cognitive abilities, such as thinking, learning, and problem-solving.

#### Subtheme 4.1. Human and brain analogies

Students associated generative artificial intelligence with human cognition, which may increase perceived trust, while potentially reducing critical evaluation of outputs.

Generative artificial intelligence is like a human because it finds solutions to problems. (P64)

Generative artificial intelligence is like a brain because it collects data and makes it meaningful. (P126) 

Generative artificial intelligence is like a wise person because it helps us learn through experience. (P58)

#### Subtheme 4.2. Developing and learning intelligence

This subtheme emphasized growth and learning over time. GenAI may be perceived as an evolving system, which could contribute to interpretations related to autonomy and intelligence.

Generative artificial intelligence is like a growing child because it develops as it learns. (P41)

Generative artificial intelligence is like a developing organism because it renews itself over time. (P327)

Generative artificial intelligence is like an evolving intelligence because it improves every day. (P79)

### Theme 5. Perception of generative artificial intelligence as a dual-purpose (beneficial–risky) technology

This theme reflects students’ awareness that generative artificial intelligence has both beneficial and potentially harmful aspects, depending on how it is used.

#### Subtheme 5.1. Benefits when used appropriately

Students indicated that generative artificial intelligence can be beneficial when used consciously. These findings suggest that students tend to evaluate GenAI conditionally, recognizing that its value may depend on responsible use.

Generative artificial intelligence is like medicine because it is useful when used correctly but harmful when used incorrectly. (P102)

Generative artificial intelligence is like a weapon because it can be beneficial or harmful depending on how it is used. (P106)

Generative artificial intelligence is like a jewel because it allows us to access valuable information quickly. (P103)

### Subtheme 5.2. Risks associated with misuse

This subtheme reflected concerns related to ethics, misuse, and reliability. Such awareness may indicate emerging critical perspectives, while also highlighting the potential need for structured AI literacy.

Generative artificial intelligence is like a cheat sheet because students use it for ready-made answers. (P101)

Generative artificial intelligence is like a trash bin because it contains both correct and incorrect information. (P185)

Generative artificial intelligence is like a boundary because it needs to be used carefully. (P191)

## Discussion

The findings of this study indicate that secondary school students’ metaphorical perceptions of generative artificial intelligence are generally positive, yet this positivity reflects a multidimensional and balanced evaluation rather than a one-sided optimism. From a theoretical perspective, this can be interpreted through Conceptual Metaphor Theory, which suggests that individuals make sense of abstract technologies by mapping them onto familiar cognitive domains [9]. Accordingly, the coexistence of positive and critical metaphors reflects structured cognitive processing rather than superficial interpretations.

Students predominantly conceptualized generative AI as a knowledge source, a teaching and guiding entity, and a supportive tool, indicating an awareness of its role in facilitating learning and enhancing access to information. This finding aligns with AI-supported learning frameworks, where AI functions as a cognitive scaffold that promotes self-regulated learning and learner autonomy [26, 27]. Consistent with prior research, generative AI is also recognized for its potential to enhance engagement, provide personalized learning, and deliver immediate feedback [5–7, 28].

At the same time, the perception of generative AI as a reflection of human intelligence and as a dual-purpose (beneficial–risky) technology suggests that students attribute both cognitive and ethical dimensions to AI. This can be explained through anthropomorphism theory, which highlights individuals’ tendency to assign human-like characteristics to intelligent systems, potentially increasing trust but also the risk of overreliance [29]. Similarly, previous metaphor-based studies report that students use both constructive and critical metaphors, reflecting concerns about dependency, misinformation, and superficial learning [1, 8, 14].

One of the prominent themes emerging from students’ metaphorical perceptions was the conceptualization of generative artificial intelligence as a comprehensive source of knowledge. Participants frequently described GenAI using metaphors such as an “encyclopedia” or a “library,” emphasizing its capacity to provide rapid access to a wide range of information. From a theoretical standpoint, these metaphors can be interpreted through Conceptual Metaphor Theory, which explains how learners map abstract technologies onto familiar knowledge structures, thereby positioning GenAI as an epistemic authority within their cognitive frameworks [9, 30, 31]. Consistent with prior research, generative AI tools such as ChatGPT have been shown to effectively support information retrieval, question answering, and immediate feedback in educational contexts [32, 33]. From a pedagogical perspective, this perceived function positions GenAI as a potentially valuable resource, particularly in settings where access to educational materials may be limited, thereby contributing to more equitable learning opportunities [4, 8, 34]. Moreover, this perception aligns with AI-supported learning and cognitive scaffolding frameworks, which emphasize the role of technology in extending learners’ access to information and supporting self-regulated learning processes. However, the literature also cautions that framing GenAI primarily as an authoritative knowledge source may foster overreliance and weaken students’ critical evaluation and source-verification skills [1, 8]. Given that generative AI systems can produce highly persuasive yet occasionally inaccurate or biased outputs, uncritical acceptance of such information poses pedagogical and ethical concerns [8].

Another key finding of this study is that students frequently conceptualized generative artificial intelligence as a teacher or guide, using metaphors such as “virtual teacher,” “philosopher,” and “guide.” These metaphors indicate that GenAI is perceived as an instructional agent capable of explaining concepts, providing direction, and supporting learning processes. Within educational theory, this positioning can be interpreted through constructivist and socio-cultural learning perspectives, where guidance and scaffolding play a central role in knowledge construction [35]. In this sense, GenAI appears to function as a digital scaffold that mediates students’ learning experiences. This perception is consistent with existing literature suggesting that generative AI can enhance personalized learning and increase student engagement by offering on-demand explanations and academic support [3, 36, 37]. Furthermore, similar metaphor-based studies have reported that learners frequently frame AI as a “teacher” or “guide,” indicating a recurring conceptual pattern across different educational contexts. However, positioning GenAI in a teaching role also raises pedagogical and ethical considerations. Although AI can function as a supplementary instructional resource, it cannot replace human educators, whose roles encompass emotional support, ethical guidance, and context-sensitive pedagogy. Prior research suggests that excessive reliance on AI-generated guidance may be associated with reduced emphasis on critical thinking and independent learning [1, 8, 38, 39]. Therefore, GenAI should be integrated as a supportive tool rather than a replacement for teachers, alongside pedagogical strategies that promote critical and reflective use [33].

Students’ metaphors also indicate that generative artificial intelligence is perceived as a multifunctional assistant that facilitates academic and everyday tasks. From a theoretical perspective, this positioning can be interpreted through cognitive offloading and distributed cognition frameworks, which explain how individuals delegate routine cognitive tasks to external tools in order to optimize mental resources [40, 41]. Metaphors such as “assistant,” “secretary,” and “homework partner” position GenAI as a tool that enhances students’ productivity by supporting activities including text drafting, summarization, problem-solving, and research processes [33]. While such delegation may support efficiency and enable greater focus on higher-order thinking, it also highlights the importance of maintaining a balance between technological assistance and independent cognitive engagement.

The perception of generative artificial intelligence as a human-like cognitive entity further illustrates the complexity of students’ meaning-making processes. Metaphors such as “human,” “brain,” and “wise person” suggest that students attribute cognitive and social characteristics to AI systems. This finding is consistent with anthropomorphism literature, which demonstrates individuals’ tendency to assign human-like qualities to intelligent systems, particularly in interactive contexts [29, 42], and has been reported across different cultural settings [38]. While such perceptions may enhance engagement and motivation, they may also influence levels of trust and critical evaluation. Given that generative AI systems operate based on probabilistic patterns rather than genuine understanding, it is important to support students in developing awareness of these limitations [32].

Finally, students’ metaphors indicate that generative artificial intelligence is perceived as a technology encompassing both benefits and potential risks. Rather than treating this as a repeated dichotomy, the findings suggest a more integrated understanding in which the perceived value of GenAI depends on how it is used and the competencies of the user. Metaphors such as “medicine” and “weapon” reflect this conditional evaluation, which aligns with the opportunity–risk perspective widely discussed in the literature [8, 33]. While GenAI offers advantages such as personalized learning, rapid content generation, and cognitive support, it also entails challenges including misinformation, academic integrity concerns, and data privacy issues [1, 32].

Overall, the findings suggest that students perceive generative artificial intelligence within a multidimensional framework that encompasses cognitive, pedagogical, and ethical dimensions. This highlights the need for AI literacy approaches that integrate technical knowledge with critical thinking, ethical awareness, and responsible use practices [30, 31].

### Limitations and future studies

This study has several limitations. First, the sample was drawn from a single region in Türkiye, which may limit the transferability of the findings to other cultural and educational contexts. Future studies including students from different regions and countries would strengthen cross-cultural understanding of adolescents’ perceptions of generative artificial intelligence. Second, data were collected using a single metaphor prompt. Although this approach is effective for exploring abstract perceptions, additional qualitative methods such as interviews or focus groups could provide deeper insight into students’ cognitive and emotional interpretations of GenAI. Finally, this study focused on metaphorical perceptions rather than actual GenAI use or levels of AI literacy. Future research could combine metaphor analysis with quantitative measures of AI literacy, ethical awareness, and digital well-being, as well as adopt longitudinal designs to examine changes in perceptions over time.

## Conclusion

This study explored secondary school students’ metaphorical perceptions of generative artificial intelligence and demonstrated that adolescents view GenAI as both a supportive cognitive tool and a source of ethical and emotional concern. Students’ metaphors reflected perceived benefits related to learning and knowledge production, alongside ambivalence regarding dependence and control. These findings underscore the importance of addressing generative artificial intelligence in secondary education through developmentally appropriate AI literacy initiatives that integrate ethical reflection, critical thinking, and emotional awareness. Understanding adolescents’ metaphorical frameworks may support the design of safer and more responsible educational approaches to GenAI use.

## Data Availability

The corresponding author will provide the datasets used and/or analyzed during the current work upon reasonable request.
